# Treatment monitoring in metastatic colorectal cancer patients by quantification and *KRAS* genotyping of circulating cell-free DNA

**DOI:** 10.1371/journal.pone.0174308

**Published:** 2017-03-22

**Authors:** Andreas W. Berger, Daniel Schwerdel, Hanna Welz, Ralf Marienfeld, Stefan A. Schmidt, Alexander Kleger, Thomas J. Ettrich, Thomas Seufferlein

**Affiliations:** 1 Department of Internal Medicine I, Ulm University, Albert-Einstein-Allee 23, Ulm, Germany; 2 Institute of Pathology, Ulm University, Albert-Einstein-Allee 23, Ulm, Germany; 3 Department of Diagnostic and Interventional Radiology, Ulm University, Albert-Einstein-Allee 23, Ulm, Germany; University of Crete, GREECE

## Abstract

Treatment of metastatic colorectal cancer (CRC) has continuously improved over the last decade. However, disease monitoring remains underdeveloped and mostly dependent on imaging e.g. RECIST 1.1 criteria. The genetic landscape of individual cancers and subsequently occurring treatment-induced evolution remain neglected in current surveillance strategies. Novel biomarkers demand minimally invasive and repetitive tracking of the cancer mutagenome for therapy stratification and to make prognostic predictions. Carcinoembryonic antigen (CEA), a routinely used tumor marker for CRC, does not meet these goals and thus prevents its use as a reliable monitoring tool. A tumor-derived fraction of circulating cell-free DNA (cfDNA), isolated from blood samples, may bypass the limitations of currently available biomarkers and could be a tool for noninvasive disease monitoring. Here, total cfDNA levels differentiated a cohort of metastatic CRC patients from healthy controls. Furthermore, we correlated cfDNA during chemotherapy of 27 stage IV patients with clinical parameters to establish its prognostic and predictive value. Indeed, cfDNA levels in chemotherapy naive patients correlate with the tumor burden and CEA values at diagnosis and increase upon disease progression during 1^st^ and 2^nd^ line treatment. Moreover, we confirm the possibility of cfDNA-based genotyping of *KRAS* to early detect the emergence of resistance during chemotherapy. These data indicate that repetitive quantitative and mutational analysis of cfDNA might complement current treatment standards but may have also limited value in some patients.

## Introduction

Despite intense efforts in the prevention and screening, colorectal cancer (CRC) remains an important contributor to cancer morbidity and mortality worldwide. In Germany, CRC is the second most common cancer among men and women. Various treatment options are available depending mostly on disease stage. In recent years, there has been a substantial improvement of survival in the metastatic situation due to novel therapeutic agents such as anti-*EGFR* antibodies in the absence of *RAS* mutations. However, valid prognostic and/or predictive biomarkers or noninvasive measures to study response or failure of a given therapeutic regimen are still missing.

The image-based Response Evaluation Criteria in Solid Tumors 1.1 (RECIST 1.1) considers changes in the longest axial tumor diameters assessed by cross section imaging and remains the gold standard to define treatment response [1]. RECIST 1.1 evaluation has several limitations and size measurements not necessarily reflect the true tumor burden [2, 3]. Furthermore, spatial heterogeneity of tumor lesions has been suggested to correlate with tumor biology and treatment resistance but is not reflected by RECIST 1.1 criteria. In addition, sole RECIST-based tumor response in metastatic CRC fails to predict survival to an individual patient [4].

Besides its impact on histological diagnosis, the molecular characterization of biopsies and resected tumor specimens enabled a prediction of disease progression and response to therapies in CRC [5]. However, there are also limitations in studying a single snapshot of a tumor due to both intertumoral and intratumoral heterogeneity and treatment associated tumor evolution. Hence, a single biopsy is likely to underestimate the complexity of the tumors genomic landscape [6]. This highlights the difficulty to determine an optimal and mutation tailored treatment process based on a single or even repetitive tumor tissue biopsy.

Blood based biomarkers for treatment stratification and therapy monitoring would be desirable tools. Analysis of carcinoembryonic antigen (CEA) is routinely used as a CRC-specific tumor marker. Even though it is elevated in multiple other malignancies and benign conditions and performs with limited sensitivity and specificity [7–9]. New minimal or noninvasive approaches to observe tumor dynamics and tumor genetics have evolved but are not yet clinically validated. One of these approaches is the analysis of circulating cell-free DNA (cfDNA) isolated from a patient´s blood sample. In the past 10 years, cfDNA has been a subject of intense research in different clinical scenarios, such as muscle training, end-stage renal failure, prenatal assessment of the unborn, stroke, surgery and trauma including myocardial infarction [10]. In clinical oncology cfDNA has been suggested as a new surrogate marker for therapy response, disease progression or early relapse [11–20].

Healthy subjects exhibit only low levels of circulating nucleic acids in their blood stream due to physiological tissue renewal. This balance can be disturbed even upon benign tumorigenesis as recently shown by our group for patients with cystic pancreatic tumors, having significantly higher cfDNA levels compared to healthy individuals. Moreover, cfDNA has the potential to discriminate patients with benign and malignant pancreatic diseases [21] and thus confirms data from previous reports. Therefore, cfDNA could be a promising tool for repeated noninvasive tumor assessment in oncology [22]. Moreover, studies have revealed a significant correlation between disease stage and the presence of tumor-associated genetic aberrations in the blood of patients with resectable [13, 23] or metastatic CRC and other early- and late-stage human malignancies [24, 25]. Tumor burden also correlates with the quantity of released cfDNA [26]. Similarly, cfDNA dynamics may predict therapy response or failure, or progression free survival (PFS) in metastatic CRC patients [27, 28]. Larger clinical trials to verify these observations are few and hampered by the limited amount of plasma samples for analysis after baseline [27]. Hence, the prognostic or predictive impact of cfDNA dynamics during treatment in metastatic CRC patients remains to be clarified.

Besides quantification, cfDNA analysis also provides the opportunity for targeted genotyping to detect the emergence of therapy resistance. The latter is considered as the most promising application of cfDNA in clinical oncology as shown in pioneering work of Diaz LA Jr et al. [11] and Misale et al. [12]. In addition, the non-invasive detection of emerging *KRAS* mutations in cfDNA from peripheral blood can help to measure resistance to *EGFR* blockade [29, 30]. Similarly, high levels of *KRAS* mutant alleles in the plasma indicate a poor outcome for patients when treated with cetuximab [19]. Bettegowda et al were able to show, that circulating tumor DNA is a broadly applicable, sensitive, and specific biomarker that can be used for a variety of clinical and research purposes in patients with multiple different types of cancer [24]. Murtaza et al. reported on sequencing of cancer exomes in serial plasma samples to track the genomic evolution of metastatic cancers during treatment. These proof-of-principle studies showed that cfDNA-based genotyping could complement current invasive biopsy approaches to identify mutations associated with acquired drug resistance in advanced cancers [31].

In this study, we hypothesized that quantitative cfDNA analysis complemented with targeted genotyping for *KRAS* under palliative chemotherapy may fulfill the requirements for implementation of cfDNA as a noninvasive monitoring tool in metastatic CRC patients during subsequent lines of palliative chemotherapy.

## Patients and methods

### Institutional review board

Prior to start of the study a positive vote from the institutional review board of Ulm University was obtained (Ethics Committee of Ulm University, Ulm, Germany, Approval numbers: 317/12, 230/14, 128/15). Participation to the study was voluntary. All patients, prior to inclusion, signed a written informed consent.

### Patient characteristics and study design

Twenty-seven patients with histologically confirmed metastatic CRC (UICC stage IV) were enrolled into a treatment surveillance cohort. All of these patients received palliative chemotherapy: 15 patients were analyzed during 1^st^ line, another 13 patients during 2^nd^ line, 3 different patients during 3^rd^ line and one patient during 4^th^ line treatment. Of note, not the same patients were analyzed during all mentioned lines of treatment. The clinical data of theses 27 patients are shown in **Table 1**, detailed patient characteristics and information about chemotherapeutic regimens are provided in **S1 Table**.

**Table 1:** Patient characteristics. Characteristics and clinical parameters of metastatic colorectal cancer patients (surveillance cohort).

Characteristic	N (%)	Characteristic	N (%)
**Patients**	27	**Resected primary tumor**	
**Age (years)**	65.4±1.8	Yes	23 (85)
**Gender**		No	4 (15)
Male	23 (85)	**Metastatic sites**	
Female	4 (15)	1	5 (19)
**Therapy lines**	32	2	12 (44)
1st line	15 (47)	>2	10 (37)
2nd line	13 (41)	***KRAS* Status**	
3rd line	3 (9)	Wild-type	16 (59)
4th line	1 (3)	Mutated	11 (41)
**Median PFS (months)**		Chemotherapy regimen*	
1st line	5.0±2.75	Oxaliplatin-based	11 (34)
2nd line	2.5±0.75	Irinotecan-based	16 (50)
3rd line	2.0±0.5	Other	5 (16)
4th line	2.75	anti*EGFR*	8 (25)
**Primary tumor site**		anti*VEGF*	17 (53)
Right-sided colon	8 (30)		
Left-sided colon	9 (33)		
Rectum	10 (37)		

* Of total 32 therapy lines; detailed characteristics available in S1 Table.

Blood samples for cfDNA analyses were taken prospectively at predefined time points (“baseline”: at least 7 days prior therapy initiation; “upon treatment”: 3.6±0.15 weeks after treatment initiation; “progression”: at radiological confirmed disease progression, ±7 days after the respective CT scan) and analyzed retrospectively. CfDNA was quantified fluorometrically. The *KRAS* status of the tumor was assessed routinely in FFPE tumor tissues at baseline (at initial diagnosis) by pyro-sequencing. Furthermore, we used droplet digital PCR (ddPCR) for *KRAS* genotyping of cfDNA at the respective time points. For the analysis of mutational concordance between tissue DNA and cfDNA we analyzed additional 23 therapy naïve patients with histologically confirmed metastatic CRC (**S3 Table**) in addition to 17 selected patients of the treatment surveillance cohort. All of these 40 tissue and blood samples were taken prior to start of 1^st^ line treatment. CT-scans were done at baseline and tumor burden was determined according to the guidelines laid out by RECIST 1.1 for measurable lesions. For restaging CT scans were performed at a mean of 2.35±0.14 months after therapy initiation and target lesions were again evaluated according to RECIST 1.1 criteria. CEA measurements were performed in parallel to cfDNA analyses at baseline, staging and restaging time points. Additionally, cfDNA was compared to a previously reported [21] cohort of 38 healthy controls (**S2 Table**) without any chronic, malignant or inflammatory diseases.

### Plasma collection

7.5ml of whole venous blood were collected in EDTA tubes (Sarstedt, Nümbrecht, Germany) by peripheral blood draw. The tubes were kept at 4°C until separation, which was carried out within one hour after collection. Whole blood was centrifuged for 10 minutes (820 x g at 4°C) and the plasma fraction was transferred in cold 2ml tubes (Eppendorf RNA/DNA LoBind micro-centrifuge tubes, Eppendorf, Hamburg, Germany). These were subsequently centrifuged again for 10 minutes (20,000 x g at 4°C) and pure plasma was recovered in fresh 2ml tubes for immediate storage at -80°C until cfDNA extraction.

### Extraction of circulating cell-free DNA

Circulating cell-free DNA was extracted from plasma using the QIAamp Circulating Nucleic Acid Kit (QIAGEN, Hilden, Germany) according to the manufacturer’s instructions. For each patient we used 2ml of plasma for cfDNA extraction and recovered cfDNA in 50μl of elution buffer. DNA was stored at 4°C until further use.

### Quantification of cell-free DNA

The total amount of cfDNA was determined by fluorometric measurement using Qubit 2.0 Fluorometer (ThermoFisher Scientific, Waltham, Massachusetts, USA). We used 2μl of DNA eluate gained through the QIAamp Circulating Nucleic Acid Kit and measured the concentration using the Qubit dsDNA HS Assay Kit (ThermoFisher Scientific, Waltham, Massachusetts, USA) according to the manufacturer’s instructions.

### Droplet digital PCR (ddPCR) analyses

Isolated cfDNA was amplified using ddPCR™ Supermix for Probes (Bio-Rad^®^, Hercules, California, USA) and the respective PrimePCR™ ddPCR™ Mutation Assay for *KRAS* p.A146T, *KRAS* p.A59T, *KRAS* p.Q61H, *KRAS* p.Q61L and the ddPCR™ *KRAS* Screening Multiplex Kit (Bio-Rad^®^, Hercules, California, USA) which covers the 7 most frequent *KRAS* mutations in colorectal cancer (*KRAS* p.G12A/G12C/G12D/G12R/G12S/G12V/G13D). 8μl/9μl of isolated cfDNA were used in each reaction and mixed with 2μl/1μl of primers/probes and 10μl of Supermix. The reaction mix was then vortexed and immediately transferred into a DG8™ Cartridge together with 70μl of Droplet Generation Oil for Probes for droplet generation in a QX200™ Droplet Generator (all: Bio-Rad^®^, Hercules, California, USA). Droplets were carefully transferred into a 96-well plate, which was sealed with PX1™ PCR Plate Sealer for subsequent amplification in a T100™ Thermal Cycler according to the manufacturer’s instructions (all: Bio-Rad^®^, Hercules, California, USA). Droplets were analyzed in QX200™ Droplet Reader (Bio-Rad^®^, Hercules, California, USA) for fluorescent measurement of FAM and HEX probes. We used reference DNA by Horizon^®^ (SW48, human, CRC stage IV) as positive (50% WT, 50% Mutant) and negative (100% WT) controls and H_2_0 as no-template-control. Thresholding was done based on positive and negative controls for each assay. False-positive-rates (FPR) were determined for each assay individually using wild-type reference DNA (Horizon^®^) in appropriate concentrations. Samples were called positive based on Poisson distribution when reaching 99% confidence level for being positive. DdPCR data were analyzed by QuantaSoft analysis software (version 1.7.4) according to the manufacturer’s instructions (Bio-Rad^®^, Hercules, California, USA).

### Assessment of *KRAS*-Status in tumor tissue at baseline

For the identification of *KRAS* mutations, H&E-stained slides from FFPE tumor samples were reviewed by a pathologist and tumor tissue was selected for analysis. Corresponding tissue from two unstained, 5-μm-thick tissue sections was removed by micro-dissection and the tissue was lysed by incubation in TEN buffer (1mM EDTA, 10mM TRIS-HCl, 0.1M NaCl, pH 8,0) including Proteinase K (20mg/ml) over night at 62°C. The resulting lysates were used for PCR reactions to amplify the regions encoding codons 12/13 in exon 2, 59 to 61 in exon 3, and 117 and 146 in exon 4 of the *KRAS* gene. For this 3μl of the lysate were added to 47μl of the PCR reaction including 25μl PCR Master Mix S (Peqlab), 0,5μl of the specific PCR primer mix (25pmol of each primer) and 21,5μl Millipore H2O. The cycling conditions were: 95°C 5 min; 2x[95°C 30sec; 62°C 30sec; 72°C 30sec]; 2x[95°C 30sec; 60°C 30sec; 72°C 30sec]; 2x[95°C 30sec; 58°C 30sec; 72°C 30sec]; 35x[95°C 30sec; 58°C 30sec; 72°C 30sec]; 72°C 10min. A fraction of the PCR reaction was separated on a 1% agarose gel to ensure the successful amplification of the *KRAS* regions. The mutation status at the different *KRAS* codons was determined by pyro-sequencing using the PyroMark Q24 sequencer (QIAGEN, Hilden, Germany). Pyro-sequencing was performed according the instructions of the manufacturer using the pyro-sequencing reagents from QIAGEN (Hilden, Germany). The sequences of all primers are available upon request.

### Statistical analyses

Results for continuous variables are presented as median ± median absolute deviation (MAD) or mean ± standard error of the mean (SEM) unless stated otherwise. Treatment groups were compared with the Student’s t-test or the Mann-Whitney U test. Comparison of categorical variables was generated by Fisher’s exact test. Survival curves were compared with the Mantel-Cox log-rank test. Correlation analyses were performed by Pearson or Spearman correlation analysis, p-values < 0.05 were considered significant. All statistical analyses were performed using GraphPad Prism version 7 (GraphPad Software, La Jolla, California, USA).

## Results

### Baseline cfDNA and CEA values

**Fig 1:**
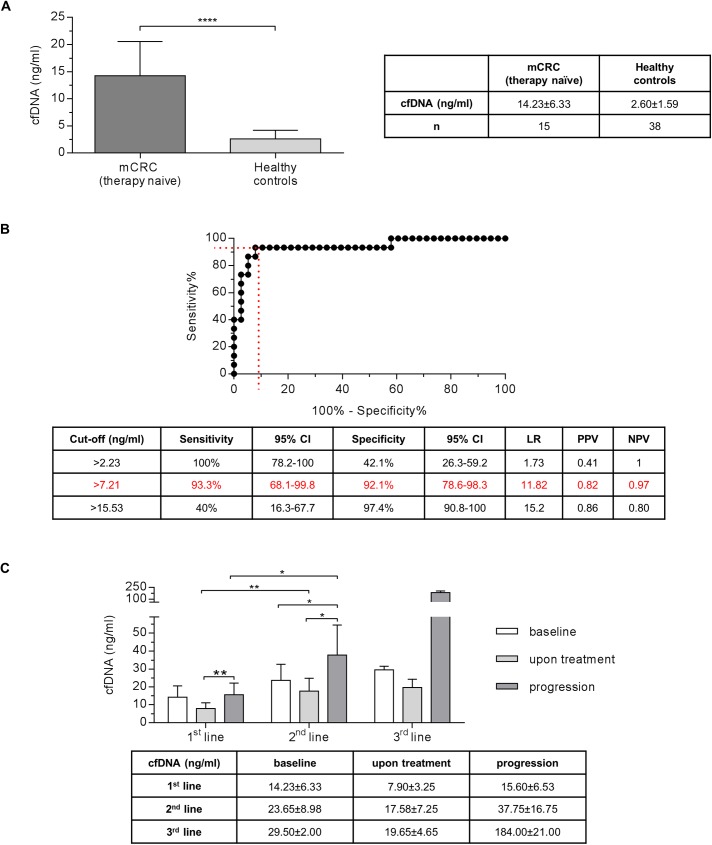
Diagnostic power of cfDNA in metastatic colorectal cancer patients and its changes under palliative treatment. **(A)** Therapy naïve patients with metastatic colorectal cancer (CRC) show significantly higher cfDNA concentration than healthy controls (Mann-Whitney, *p<0*.*0001*). **(B)** Receiver operating characteristic (ROC) of cfDNA level as a test parameter to discriminate patients with metastatic CRC from healthy controls. **(C)** CfDNA levels over the time course of therapy in 1^st^, 2^nd^ and 3^rd^ line treatment. The amount of cfDNA increases at time-point of progression in 1^st^ and 2^nd^ line (Mann-Whitney, *p<0*.*01*, *p<0*.*05*, respectively). CI = confidence interval, LR = likelihood ratio, PPV = positive predictive value, NPV = negative predictive value. Level of significance: * = p<0.05, ** = p<0.01, *** = p<0.001, **** = p<0.0001.

First, we examined the level of cfDNA in therapy naïve UICC stage IV metastatic CRC patients prior to systemic treatment. The median baseline cfDNA value was 14.23±6.33ng/ml. There was no significant difference between the cfDNA concentrations of right- and left-sided primary CRCs (15.76±9.15ng/ml vs.14.23±6.23ng/ml, *p = 0*.*9495*, data not shown). However, there was a significant difference in cfDNA content between patients with metastatic CRC and healthy individuals with a median value of 2.60±1.59ng/ml (*p<0*.*0001*, **Fig 1A**). Test performance analyzed by Receiver operating characteristic (AUC = 0.94, **Fig 1B**) showed reasonable sensitivity (93.3%) and specificity (92.1%) when using a threshold of 7.21 ng cfDNA per ml of plasma to discriminate the two cohorts. Total cfDNA levels were independent of age (data not shown) or sex (therapy naïve mCRC cohort *p = 0*.*400*; healthy controls *p = 0*.*5297*, data not shown). CEA was elevated [≥ 2.5μg/l] at baseline in 86.7% of the cases with a median baseline level of 19.80±17.9μg/l (data not shown).

### cfDNA levels during palliative 1^st^ and 2^nd^ line chemotherapy

Next, we examined cfDNA levels and their changes during 1^st^ and 2^nd^ line chemotherapy. CfDNA concentration was determined prior to start of chemotherapy in each therapy line (1^st^ or 2^nd^ line treatment), after 3.96±0.28 weeks of treatment (termed “upon treatment” time point) and upon radiologically confirmed disease progression (termed “progression”).

Under 1^st^ line palliative chemotherapy reduced cfDNA levels were observed after 4 weeks of chemotherapy dropping from 14.23±6.23ng/ml to 7.9±3.25ng/ml (*p = 0*.*0515*). At the time of disease progression, cfDNA levels were significantly increased in the 1^st^ line setting to 15.6±6.53ng/ml compared to the “upon treatment” time point (*p<0*.*01)* (**Fig 1C**). During 2^nd^ line treatment, we observed increased cfDNA levels compared to the 1^st^ line situation. Though this observation was not statistical significance at baseline (*p = 0*.*2005*) but upon treatment (*p<0*.*01*) and at progression (*p<0*.*05*) a significant difference could be observed (**Fig 1C**). Similarly, there was no significant difference (*p = 0*.*798*) between cfDNA levels at baseline of 2^nd^ line (23.65±8.98ng/ml) and upon treatment of 2^nd^ line (7.58±7.25ng/ml). However, upon disease progression median cfDNA value significantly increased to 37.75±16.75ng/ml (vs. “baseline”: *p<0*.05; and vs. “upon treatment”: *p<0*.05, **Fig 1C**). CfDNA dynamic during 3^rd^ line chemotherapy was overall higher with a similar pattern compared to the respective states prior 1^st^ and 2^nd^ line treatment. Intriguingly, a substantial increase in cfDNA levels was observed “at progression” in 3^rd^ line but again not significant (*p = 0*.*2000*, **Fig 1C**).

### Correlation of cfDNA and tumor burden (RECIST 1.1)

**Fig 2:**
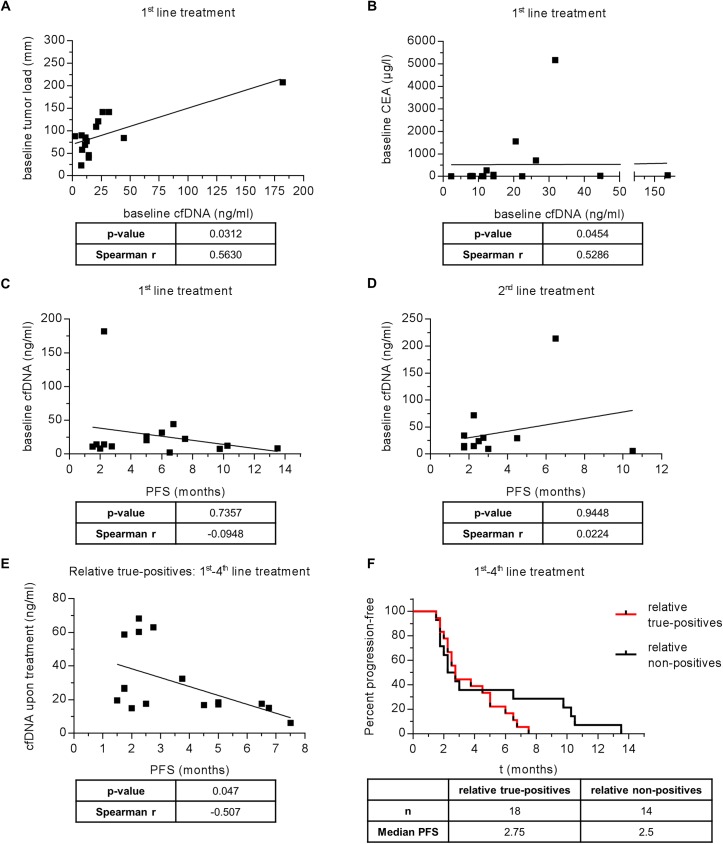
Correlation of respective cfDNA and CEA values with clinical parameters in therapy naïve and pretreated patients. Correlation of baseline cfDNA levels in therapy naïve metastatic CRC patients with **(A)** baseline tumor load (RECIST 1.1) and **(B)** baseline CEA level **(C)** with progression-free survival (PFS). **(D)** Correlation of baseline cfDNA levels in pretreated metastatic CRC patients with progression-free survival (PFS). **(E)** + **(F)**: patients defined as relative true-positives concerning cfDNA level at baseline (>95% of highest control). **(E)** Correlation of cfDNA level upon treatment with PFS of relative true-positive group. **(F)** Kaplan-Meier-plot showing PFS of relative true-positives vs relative non-positives.

Tumor burden of all individuals in our study was determined according to the guidelines laid out by RECIST 1.1 for measurable lesions. Baseline cfDNA levels in therapy naïve patients significantly correlated with the respective tumor burden (*p<0*.*05*, r = 0.563). (**Fig 2A**). However, such a correlation could not be observed in subsequent therapy lines (*p = 0*.*5746*, r = 0.1706, data not shown). Interestingly, cfDNA levels did not correlate with tumor burden according to RECIST 1.1 criteria at disease progression under 1^st^ line (*p = 0*.*3538*, data not shown) and 2^nd^ line (*p = 0*.*2769*, data not shown) treatment.

### Predictive value of cfDNA for progression-free survival (PFS)

To determine whether these findings would correlate with PFS we analyzed all data obtained in the 1^st^ and 2^nd^ line setting. No significant influence on PFS for cfDNA levels both at baseline (**Fig 2C**) and upon treatment (**S1A Fig**) in the 1^st^ line and 2^nd^ line setting could be observed (**Fig 2D**, **S1C Fig**). We then normalized cfDNA baseline values of our treatment surveillance cohort to 95% of the highest cfDNA level in our healthy control cohort to identify relative true-positives. By excluding the relative non-positives from the analysis, a significant correlation between the cfDNA levels upon treatment and PFS can be demonstrated (**Fig 2E,** *p = 0*.*047*, r = -0.507). The respective cfDNA levels at baseline did not correlate with PFS (*p = 0*.*600*, data not shown). Similarly, the “relative true-positives” does not significantly differ from the “relative non-positives” group in the Kaplan Mayer analysis to assess PFS (**Fig 2F,** *p = 0*.*2584*).

### Correlation of cfDNA and CEA levels

Corresponding cfDNA levels and CEA measurements were available from all patients at corresponding time points. There was a significant, positive correlation between baseline cfDNA levels and CEA values in therapy naïve patients (*p = 0*.*0454*, r = 0.5286, **Fig 2B**), while no correlation was observed at later time points during 1^st^ line (”upon treatment”: *p = 0*.*3310*;”progression”: *p = 0*.*9132*, data not shown) or in the 2^nd^ line setting (“baseline”: *p = 0*.*6960*, “upon treatment”: *p = 0*.*4511*, progression: *p = 0*.*2316*, data not shown). Interestingly, there was also no correlation between CEA baseline levels and PFS in the 1^st^ line (*p = 0*.*7602*, data not shown) or the 2^nd^ line setting (*p = 0*.*4035*, data not shown). In contrast, CEA levels upon treatment correlated with PFS in the 1^st^ (*p = 0*.*0280*, r = -0.6410, **S1B Fig**) but not during 2^nd^ line treatment (*P = 0*.*6638*, **S1D Fig**).

**Fig 3:**
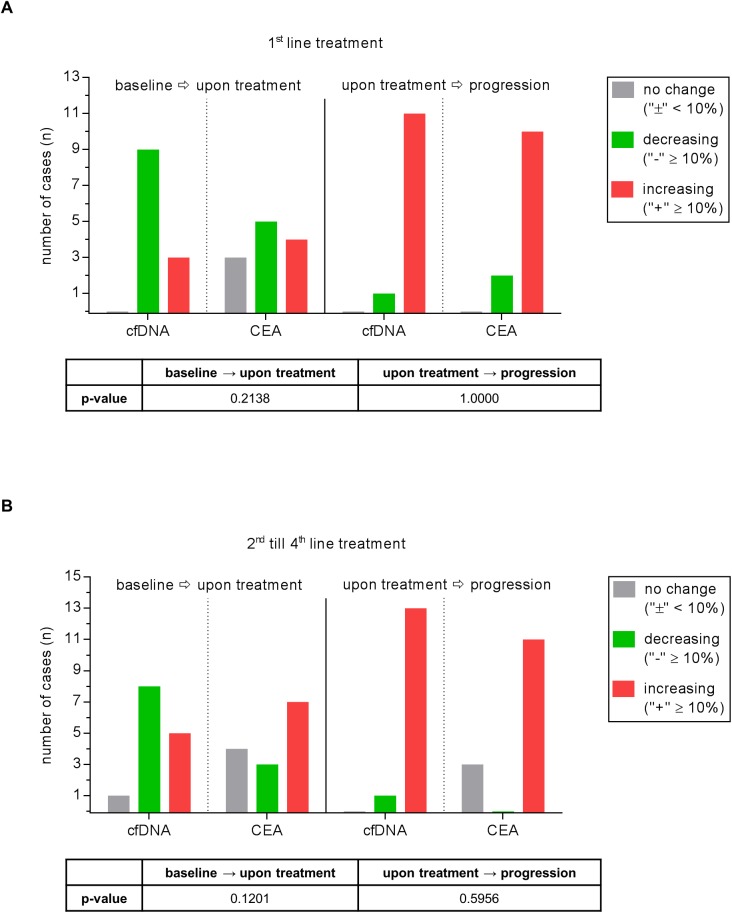
Comparison of the power of cfDNA and CEA course to reflect the course of disease. Values are depicted for both therapy naïve patients **(A)** and patients during further therapy lines **(B)**. A change in cfDNA amount of ≥10% is considered as “increase” /”decrease”. A change in value <10% is defined as “no change”.

Next, we investigated the power of cfDNA and CEA to mirror disease course. A 10% change in the CEA or cfDNA value across various time points was determined as a clinically meaningful difference [28]. In the 1^st^ line situation cfDNA decreased in 75% of cases under therapy while CEA decreased in only 56%. At the time of disease progression there was an increase in cfDNA levels in 92% of cases and in CEA levels in 83% (**Fig 3A**). This was even more pronounced in advanced treatment lines (2^nd^ - 4^th^ line). Here we observed decreasing cfDNA levels under therapy in 57% of cases versus 21% for CEA and increasing cfDNA levels towards disease progression in 93% versus 79% for CEA (**Fig 3B**).

### *KRAS* genotyping of tumor tissue and cfDNA in therapy naïve patients

**Fig 4:**
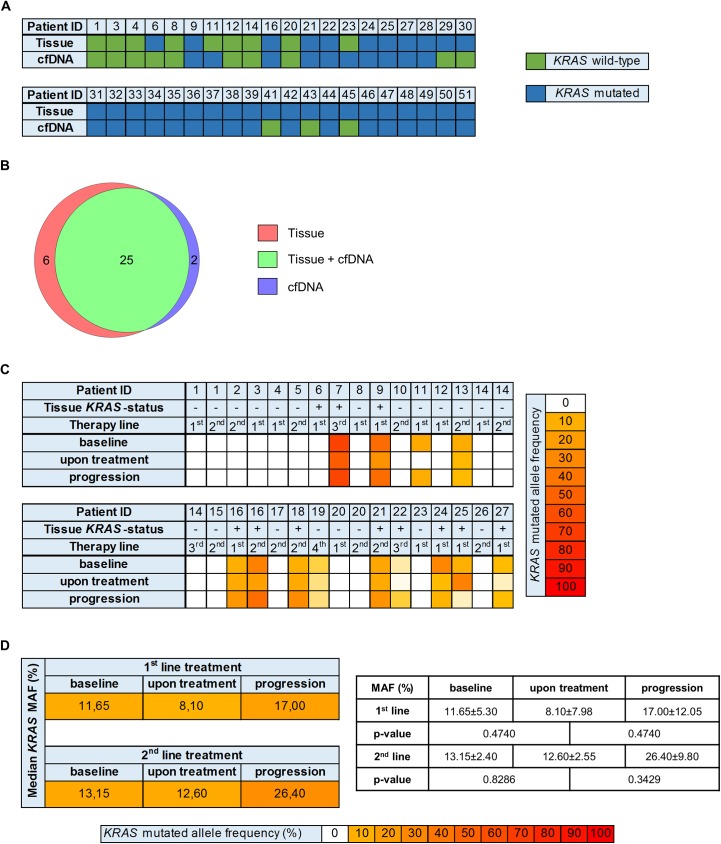
*KRAS* genotyping of cfDNA. **(A)** *KRAS* mutational status per patient for tissue and cfDNA analysis at time-point of initial diagnosis (n = 40, all: therapy naïve). **(B)** Venn diagram showing that 25 *KRAS* mutations, detected in tumor tissue could also be found in cfDNA, 6 were only present in tumor tissue and 2 only in cfDNA. **(C)** *KRAS* mutated allele frequencies in cfDNA under treatment per patient and therapy line. **(D)** Median *KRAS* mutated allele frequencies (MAF) in 1^st^ and 2^nd^ line treatment group at the respective time-points.

First, we applied pyro-sequencing technologies of hot-spot regions in the *KRAS* gene at initial diagnosis of metastatic CRC. Specifically, we assessed the *KRAS* status in exon 2 (codons 12/13), exon 3 (codon 59 and 61) and exon 4 (codon 117 and 146) and identified 77.5% (31 out of 40) to carry any *KRAS* mutation within these hotspots. These data were complemented using digital droplet PCR techniques (ddPCR) from cfDNA of the respective patients. Such analysis identified 2 additional patients to be *KRAS* mutated based on cfDNA genotyping suggesting heterogeneity. Vice versa, cfDNA analysis missed 6 patients with *KRAS* mutations. Details are shown in **Fig 4A and 4B, S3 and S4 Tables**. To specify these numbers, we calculated the overall per patient concordance of *KRAS* mutational status between cfDNA and tumor tissue with 80% in therapy naïve patients (**Fig 4B**).

**Fig 4C** shows *KRAS* mutated allele frequencies (MAF) for the treatment surveillance cohort during treatment. 6 of the 15 therapy naïve patients (40.0%) showed *KRAS* mutations. The median MAF of the patients with mutated *KRAS* decreased from baseline (11.65±5.30%) to the “upon treatment” time point (8.10±7.98%, *p = 0*.*4740*) and increased again at progression (17.00±12.05%, *p = 0*.*4740*) in the 1^st^ line treatment group (**Fig 4D**).

### *KRAS* genotyping of tumor tissue and cfDNA in advanced therapy lines

In pretreated patients, 4/13 (30.8%) patients under 2^nd^ line treatment and 2/3 (66.7%) patients under 3^rd^ line treatment and 1/1 (100%) patient under 4^th^ line treatment exhibited mutated *KRAS* in cfDNA at baseline (**Fig 4C**). The median MAF for *KRAS* of the patients under 2^nd^ line treatment decreased from baseline (13.15±2.40%) to the “upon treatment” time point (12.60±2.55%, *p = 0*.*8286*) and increased again at progression (26.40±9.80%, *p = 0*.*3429*) (**Fig 4D**).

### *KRAS* genotyping of cfDNA during therapy

It has been suggested that amongst other causes *KRAS* mutations emerge during anti-*EGFR* treatment and mediate acquired resistance. Therefore, we analyzed the respective parameters in some of our patients (**S2 Fig**).

In one patient (**S2A Fig**), the primary tumor tissue was *KRAS* exon 2–4 wild type, similarly cfDNA analysis confirmed the *KRAS* wild type status at baseline before initiating of 1^st^ line therapy. In this patient, the drop in cfDNA correlated with a sustained response during treatment. There was also a correlation of radiologically confirmed disease progression with a rise in cfDNA. The CEA value of this patient also dropped during treatment, but failed to increase upon progression. However, a *KRAS* Exon 2 mutation was detectable at the time point of radiologically confirmed disease progression.

In another patient (**S2B Fig**), where the tumor also was determined as *KRAS* exon 2–4 wild type, we detected a *KRAS* Exon 3 mutation by cfDNA analysis already at baseline (prior 1^st^ line therapy), namely Q61H. Since tissue analysis was the standard of *KRAS* assessment at that time the patient received a combination of FOLFIRI plus panitumumab. Interestingly, the *KRAS* mutation was not any more detectable one month after therapy initiation, possibly due to the treatment with cytotoxic agents (irinotecan-based chemotherapy). However, the same mutation was detectable again in cfDNA about 5 months later, more than 2 months prior to radiologically confirmed disease progression. The results of this patient indicate the potential usefulness of cfDNA-based *KRAS* screening during treatment for early detection of disease progression in contrast to sole cfDNA quantification.

Another patient (**S2C Fig**) received an anti-*EGFR* antibody as single agent as 4^th^ line treatment. The tumor tissue was defined as *KRAS* exon 2–4 wild type prior to 1^st^ line therapy and no further tissue analyses were done according to standard clinical practice. However, we detected a *KRAS* exon 3 mutation (Q61H) over the whole period of 4^th^ line treatment in our retrospective analysis. Consequently, there was disease progression already at the first staging after 2 months of treatment. As expected from the data described above, disease progression was accompanied by an increase in total cfDNA value.

## Discussion

Data from this study and from other groups [32] demonstrate that cfDNA can be faithfully isolated and analyzed in patient samples. First described in 1948, cfDNA has been analyzed for many years to test its potential as a noninvasive biomarker in cancer patients [13, 31, 33–37]. Patients with malignant tumors carry significantly more cfDNA compared to those with benign or premalignant lesions or healthy individuals, for example in pulmonary or gastrointestinal diseases [21, 38–42]. In this study, we confirm this concept in patients with metastatic CRC compared to healthy individuals. Accordingly, cfDNA may be considered as a useful and unique biomarker in malignancy. However, the role of cfDNA as a noninvasive monitoring tool over the continuum of care is still largely elusive and only limited data are available, in particular in advanced therapy lines [27, 28, 30, 43].

For dynamic cfDNA analysis we focused on three time points that appear clinically relevant: baseline, upon treatment and at disease progression as determined by imaging. In this study, cfDNA concentration and tumor burden as determined according to the guidelines laid out by RECIST 1.1 for measurable lesions at baseline prior to 1^st^ line treatment positively correlated in therapy naïve patients (**Fig 2A**) as well as cfDNA and CEA (**Fig 2B**), which is in line with previously reported data [28]. Therefore, cfDNA quantification in therapy naïve patients allows to determine the tumor load in an easy noninvasive procedure. But comparisons between the various studies are difficult to make, not at least due to a lack of standardized procedures and technologies for isolation and quantification of cfDNA, like BEAMing, several quantitative real-time PCR protocols [27], ddPCR technologies [21, 30] or digital genomic assays, for example Safe-SeqS [28], resulting in a limited generalizability. Interestingly, cfDNA levels in our study did not correlate with tumor burden according to RECIST 1.1 criteria at disease progression under 1^st^ line and 2^nd^ treatment (**S1E and S1F Fig**). This may be caused by the fact, that RECIST 1.1 dependent disease progression is defined by a predefined increase in diameter but also by the occurrence of new metastases. When new metastases define disease progression this is not reflected by the diameter. In those cases, cfDNA level may increase due to high cellular turnover although the RECIST 1.1 diameter is not increasing. Nevertheless, the observed correlations between tumor load (RECIST 1.1), CEA and baseline cfDNA in therapy naïve patients could not be confirmed under therapy and in later therapy lines. However, an increase in cfDNA correlates well with disease progression either in 1^st^ or in 2^nd^ line treatment and cfDNA levels rise during the therapeutic course of metastatic CRC.

Moreover, it has not been conclusive, if cfDNA values at baseline and cfDNA changes under therapy are prognostic. In our study, total cfDNA values both prior 1^st^ line treatment and 2^nd^ line treatment did not show a significant correlation to PFS in the respective therapy line. Furthermore, cfDNA fold change in all therapy lines did not have a significant correlation with PFS. To further examine the prognostic value of cfDNA we normalized cfDNA baseline values of our treatment surveillance cohort to 95% of the highest cfDNA level in our healthy control cohort and thereby identified the relative true-positives, which showed a significant correlation between the cfDNA levels upon treatment and PFS (**Fig 2E**, *p = 0*.*047*, r = -0.507), in line with previously published reports [27, 28]. Similarly, CEA values upon treatment significantly correlated with PFS in the 1^st^ line situation (**S1B Fig**, *p = 0*.*0280*, r = -0.641). Accordingly, CEA seemed to outperform cfDNA in the prediction of a highly relevant endpoint (PFS) in the 1^st^ line setting. This fact may render the use of a more complicated and expensive biomarker rather redundant and needs further clarification. An assumed multivariate model would likely show limited to no value of total cfDNA quantification as a prognostic marker. Moreover, it is important to state, that the reliance of a dichotomized rather than continuous biomarker, which could help to prove the variability in calls is relevant. This is especially important to gain assumptions from longitudinal changes between the respective time points of cfDNA measurement (baseline, therapy and disease progression).

Nevertheless, our data suggest that quantitative cfDNA analysis in metastatic CRC shows some important strengths, like estimation of tumor load in therapy naïve patients but also some weaknesses. In particular, the lack of standardized procedures in cfDNA isolation, quantification and genomic analysis, and the lack of validated cut-offs for cfDNA changes, makes it difficult to assess the correlation of such changes with PFS or tumor response across clinical trials. The value of total cfDNA quantification and its impact as a predictive and prognostic tool is also limited due to several individual variations in the cellular turnover under treatment with subsequent cfDNA release. Several mechanisms of tumor evolution under treatment also affect the total amount of cfDNA release, which limits the individual comparison of total cfDNA assessment and the derivation of prognostic and/or predictive information. For sure, also the low number of patients in our study limits its meaningfulness.

Besides the quantitative assessment of cfDNA during the course of several chemotherapeutic regimens we focused on the molecular characterization of cfDNA by targeted genotyping of *KRAS* using droplet digital PCR (ddPCR), an established technology for this application [21, 30]. *KRAS* genotyping of cfDNA is not considered as a standard in metastatic CRC patients undergoing treatment with anti-*EGFR* antibodies. Primarily, it should be clarified how representative *KRAS* genotyping in cfDNA is in comparison to tumor tissue assessment, which is the gold standard in deciding regarding anti-*EGFR* treatment. In the retrospective analysis of our cohort, tissue *KRAS* mutational status at baseline could be confirmed in cfDNA in 80% of the therapy naïve patients (**Fig 4A and 4B**), highlighting the potential of this promising tool for noninvasive molecular assessment and underlining other concordance analyses of cfDNA and tumor tissue [13, 31]. The high concordance status provides the prerequisite for molecular tumor characterization on cfDNA level, which was fulfilled in our study cohort. Of interest, in 2 patients tumor tissue genotyping for *KRAS* revealed a wild type situation, while the respective baseline cfDNA harbored *KRAS* mutations. In contrast, 6 patients were confirmed as *KRAS* mutated in tumor tissue, while the corresponding cfDNA genotyping for *KRAS* showed a wild type situation. To understand the possible path forward, if they are false positives or true positives likely an outcome study is needed. The fact that different methodologies were used to assess the presence of *KRAS* mutations in cfDNA (ddPCR) and in the respective tumor tissue (pyro-sequencing) could potentially account for some discordance in our study. Moreover, while total cfDNA quantification is not suitable to study both inter- and intratumoral heterogeneity, cfDNA targeted genotyping, as it was performed for *KRAS* within our study, is indeed a means to overcome reports about single biopsy bias.

Many studies focused on *KRAS* assessment of cfDNA during the last years and demonstrated the occurrence and recurrence of mutated *KRAS* alleles under anti-*EGFR* treatment [11, 12, 19, 27–32, 37, 43]. However, in the clinical scenario it remains as yet unclear how to deal with these results. On the one hand, what to do, if inconsistent results are occurring between tissue and cfDNA at baseline and on the other hand how to proceed when *(K)RAS* mutations occur under *EGFR*-blockade. As shown by our case study (**S2A–S2C Fig**), *KRAS* mutated clones can indeed disappear under a combination of chemotherapy plus anti-*EGFR* antibody and become again detectable upon disease progression. Therefore, in some cases obviously combination chemotherapy is also capable of suppressing *KRAS* mutated clones in CRC.

Our data indicate that combined cfDNA quantification and targeted genotyping of cfDNA in metastatic CRC patients can provide some useful information for treatment monitoring and noninvasive early detection of a disease progression under a given therapeutic regimen which underlines previously reported data [11, 12, 19, 27–32, 37, 43]. Still several, in particular technical, obstacles have to be overcome. Therefore, cfDNA as a novel means of assessing tumor progression was already being integrated into many clinical trials to validate its significance and to enable technical standardization and to potentially use it for treatment stratification. Nevertheless, robust data are not yet available.

## Conclusion

In summary, the most important findings of our study are that quantitative cfDNA measurement and targeted cfDNA genotyping for *KRAS* by ddPCR provides an easy to perform and promising approach for noninvasive treatment monitoring, in particular for disease progression but not treatment response in patients with metastatic CRC. Furthermore, baseline cfDNA quantification prior 1^st^ line treatment positively correlates with tumor burden. This finding only applies to therapy naïve patients and could complement determination of the real tumor burden with a potential impact on the choice of the therapeutic regimen. Our data of this univariate analysis show, that serial cfDNA measurements provide the possibility for dynamic disease monitoring and can complement chemical laboratory (CEA) and radiological (RECIST 1.1) data for treatment monitoring in metastatic CRC patients. Upon both 1^st^ and 2^nd^ line treatment a cfDNA increase indicates a disease progression underlining its value as a monitoring tool. cfDNA true positives significantly correlated with progression free survival, highlighting the impact of cfDNA as a prognostic tool in mCRC. Moreover, cfDNA *KRAS* status is highly concordant with tissue results in therapy naïve patients. Our case reports indicate the usefulness but also the limitations of *KRAS* mutational screening prior therapy initiation, under therapy and prior to later therapy lines when using *EGFR* blocking strategies.

## Supporting information

S1 Fig Correlation of respective cfDNA and CEA values with clinical parameters in therapy naïve and pretreated patients. Correlation of progression-free survival (PFS) under 1^st^ line treatment with **(A)** cfDNA level upon treatment **(B)** and CEA level upon treatment. Correlation of progression-free survival (PFS) under second line treatment with **(C)** cfDNA level upon treatment and **(D)** CEA level upon treatment. Correlation of cfDNA level and tumor load at progression in 1^st^ line **(E)** and 2^nd^ line treatment **(F)**.
(TIF)

S2 Fig Case reports. Case reports: Tracking of cfDNA, CEA, mutated KRAS allele levels over the time course of treatment of patient **(A)** 11, **(B)** 19 and **(C)** 23. Selected CT-scans and corresponding RECIST 1.1 classification are depicted below the respective graphs. BL = baseline, SD = stable disease, PR = partial remission, PD = progressive disease.
(TIF)

S1 Table Detailed characteristics of metastatic colorectal cancer patients (surveillance cohort). (PDF)

S2 Table Patient characteristics of healthy control cohort. (PDF)

S3 Table Detailed characteristics of *KRAS* concordance cohort. (PDF)

S4 Table Detailed mutational characteristics of metastatic colorectal cancer patients (surveillance cohort). (PDF)
