# Supplementary figures and images for: Treatment monitoring in metastatic colorectal cancer patients by quantification and KRAS genotyping of circulating cell-free DNA
Source: PLoS One. 2017 Mar 22;12(3):e0174308. doi: 10.1371/journal.pone.0174308 (PMC5362218; doi:10.1371/journal.pone.0174308)

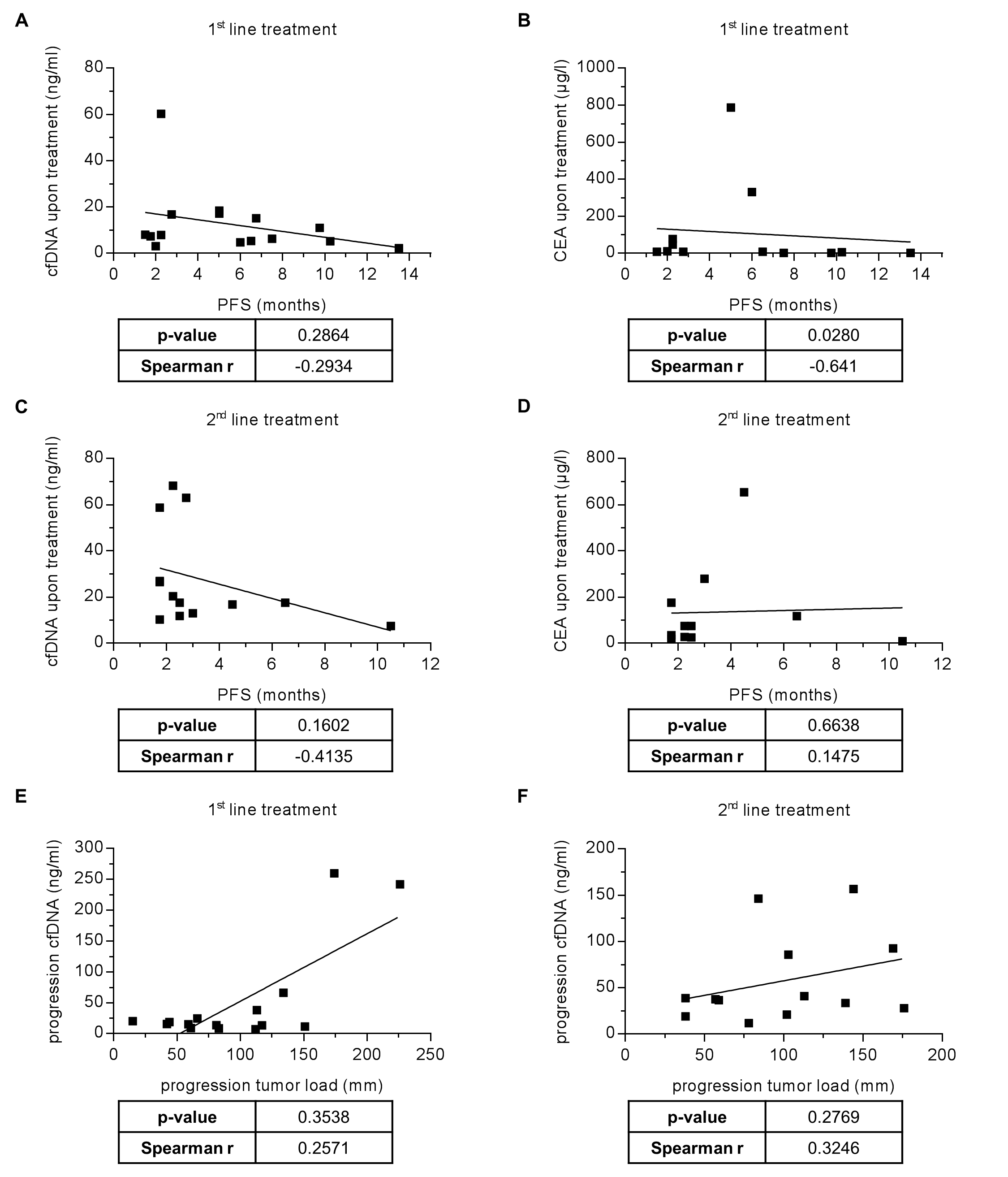

Supplement: S1 Fig — Correlation of progression-free survival (PFS) under 1st line treatment with (A) cfDNA level upon treatment (B) and CEA level upon treatment. Correlation of progression-free survival (PFS) under second line treatment with (C) cfDNA level upon treatment and (D) CEA level upon treatment. Correlation of cfDNA level and tumor load at progression in 1st line (E) and 2nd line treatment (F). (TIF) [file pone.0174308.s001.tif]

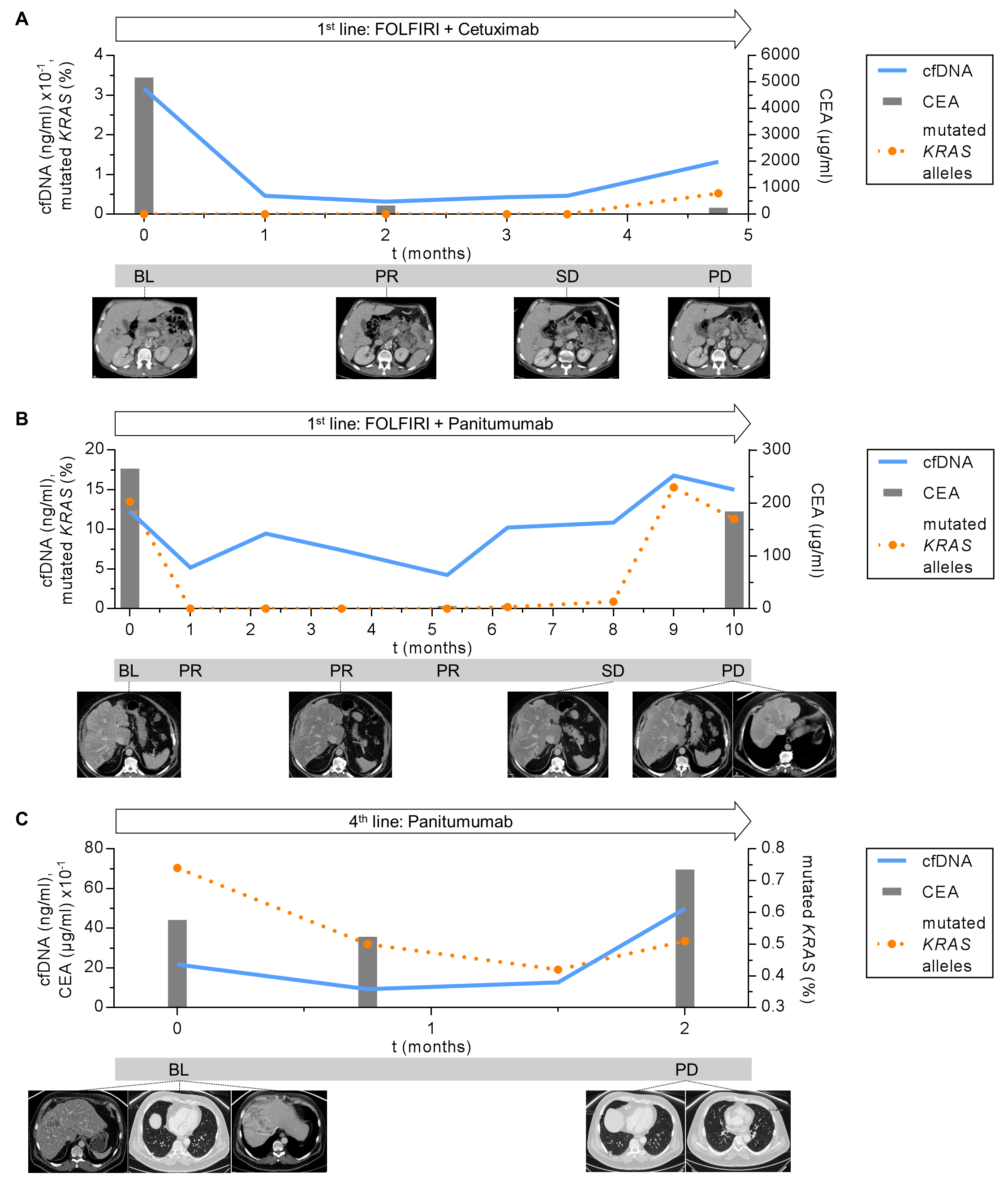

Supplement: S2 Fig — Case reports: Tracking of cfDNA, CEA, mutated KRAS allele levels over the time course of treatment of patient (A) 11, (B) 19 and (C) 23. Selected CT-scans and corresponding RECIST 1.1 classification are depicted below the respective graphs. BL = baseline, SD = stable disease, PR = partial remission, PD = progressive disease. (TIF) [file pone.0174308.s002.tif]
