# Supplementary material for: Treatment monitoring in metastatic colorectal cancer patients by quantification and KRAS genotyping of circulating cell-free DNA
Source: PLoS One. 2017 Mar 22;12(3):e0174308. doi: 10.1371/journal.pone.0174308 (PMC5362218; doi:10.1371/journal.pone.0174308)
Supplement: S2 Table — (PDF) [file pone.0174308.s004.pdf]

ID	Sex	Age (years)	cfDNA (ng/ml)
**1**	m	28	4,675
**2**	f	32	4,575
**3**	m	37	3,2
**4**	f	62	3,5
**5**	m	63	4,175
**6**	f	25	2,525
**7**	f	24	2,2175
**8**	m	27	2,675
**9**	m	30	2,95
**10**	f	30	6,75
**11**	f	31	6,5
**12**	m	29	4,65
**13**	f	28	7,8
**14**	m	41	16,8
**15**	f	27	6,55
**16**	f	32	4,3
**17**	m	34	4,225
**18**	m	78	2,44
**19**	m	68	2,7
**20**	m	40	0,58
**21**	m	60	3,5375
**22**	f	62	0,351
**23**	m	50	9,6
**24**	m	70	1,95
**25**	f	66	1,98
**26**	f	72	1,97
**27**	f	63	3,55
**28**	f	65	1,77
**29**	f	62	0,76
**30**	f	73	0,8425
**31**	m	69	1,04
**32**	f	63	1,0325
**33**	f	68	0,9
**34**	f	58	0,66
**35**	m	67	0,93
**36**	m	64	0,99
**37**	f	77	2,27
**38**	m	67	1,26
