# Supplementary material for: Treatment monitoring in metastatic colorectal cancer patients by quantification and KRAS genotyping of circulating cell-free DNA
Source: PLoS One. 2017 Mar 22;12(3):e0174308. doi: 10.1371/journal.pone.0174308 (PMC5362218; doi:10.1371/journal.pone.0174308)
Supplement: S4 Table — (PDF) [file pone.0174308.s006.pdf]

ID	Tissue *KRAS*-status (initial diagnosis)	cfDNA *KRAS*-status (baseline)	Therapy line	*KRAS* mutated allele frequency (%)		
				baseline	upon treatment	progression
**1**	wt	wt	1st	0	0	0
			2nd	0	0	0
**2**	wt	wt	2nd	0	0	0
**3**	wt	wt	1st	0	0	0
**4**	wt	wt	1st	0	0	0
**5**	wt	wt	2nd	0	0	0
**6**	G13D	wt	1st	0	0	0
**7**	G12R	codon 12/13	3rd	66.2	53.3	64.8
**8**	wt	wt	1st	0	0	0
**9**	A146T	A146T	1st	44.7	25.0	44.4
**10**	wt	wt	2nd	0	0	0
**11**	wt	Q61H	1st	13.5	0	11.3
**12**	wt	wt	1st	0	0	0
**13**	wt	codon 12/13	2nd	11.5	4.3	3.6
**14**	wt	wt	1st	0	0	0
			2nd	0	0	0
			3rd	0	0	0
**15**	wt	wt	2nd	0	0	0
**16**	G12D	codon 12/13	1st	9.8	12.4	22.7
			2nd	33.9	17.5	44.0
**17**	wt	wt	2nd	0	0	0
**18**	codon 12	codon 12/13	2nd	10.0	12.4	28.4
**19**	wt	Q61H	4th	0.7	0.5	0.5
**20**	wt	wt	1st	0	0	0
			2nd	0	0	0
**21**	G12D	codon 12/13	2nd	14.8	12.8	24.4
**22**	G12D	codon 12/13	3rd	0.3	0.1	0.8
**23**	wt	wt	1st	0	0	0
**24**	G12R	codon 12/13	1st	31.1	3.8	7.4
**25**	G12D	codon 12/13	1st	9.6	33.3	0.3
**26**	wt	wt	2nd	0	0	0
**27**	G12V	codon 12/13	1st	3.1	0.3	2.2

wt=wild-type for tested hot-spots
