# Supplementary material for: Treatment monitoring in metastatic colorectal cancer patients by quantification and KRAS genotyping of circulating cell-free DNA
Source: PLoS One. 2017 Mar 22;12(3):e0174308. doi: 10.1371/journal.pone.0174308 (PMC5362218; doi:10.1371/journal.pone.0174308)
Supplement: S1 Table — (PDF) [file pone.0174308.s003.pdf]

ID	Sex	Age (years)	Primary tumor	Primary tumor resected	Metastases		Stage	Chemotherapeutic regimen		
					Sites	Localization		Therapy line	Chemo-therapy	Biolo-gical
**1**	m	79	rectum	yes	1	HEP	UICC IV	1st	Iri/5-FU/LV	BV
								2nd	Ox/5-FU/LV	BV
**2**	m	57	rectum	yes	2	HEP, PUL	UICC IV	2nd	Iri/5-FU/LV	Afl
**3**	f	53	right	no	2	HEP , LYM	UICC IV	1st	Iri/5-FU/LV	Cet
**4**	f	80	right	yes	>2	HEP, PUL, LYM	UICC IV	1st	5FU/LV	BV
**5**	m	59	rectum	yes	1	HEP	UICC IV	2nd	Ox/5-FU/LV	-
**6**	m	70	rectum	yes	2	PUL, PLEU	UICC IV	1st	Iri/5-FU/LV	BV
**7**	m	78	right	yes	2	HEP, PUL	UICC IV	3rd	Ox/5-FU/LV	BV
**8**	m	75	left	yes	1	HEP	UICC IV	1st	Iri/5-FU/LV	Cet
**9**	m	78	right	yes	>2	HEP, PUL, PLEU, LYM	UICC IV	1st	5FU/LV	BV
**10**	m	57	left	yes	>2	HEP, PUL, OSS	UICC IV	2nd	Ox/5-FU/LV	BV
**11**	m	57	left	yes	2	HEP, LYM	UICC IV	1st	Iri/5-FU/LV	Pan
**12**	m	67	rectum	yes	2	LYM, PUL	UICC IV	1st	Iri/5-FU/LV	BV
**13**	m	75	right	yes	>2	HEP, PUL, LYM, SKI	UICC IV	2nd	Ox/5-FU/LV	BV
**14**	m	69	left	yes	2	HEP, PER	UICC IV	1st	Iri/5-FU/LV	Cet
								2nd	Ox/5-FU/LV	-
								3rd	Iri	Pan
**15**	m	61	rectum	no	2	HEP, PUL	UICC IV	2nd	Ox/5-FU/LV	BV
**16**	m	62	left	no	>2	ADR, HEP, LYM, PUL	UICC IV	1st	Ox/Iri/ 5-FU/LV	-
								2nd	Iri/5-FU/LV	BV
**17**	m	70	right	yes	2	HEP, PUL	UICC IV	2nd	Cap	BV
**18**	m	60	rectum	yes	>2	HEP, LYM, SKI	UICC IV	2nd	Ox/5-FU/LV	-
**19**	m	58	left	no	>2	HEP, PUL, LYM	UICC IV	4th	-	Pan
**20**	m	71	rectum	yes	1	LYM	UICC IV	1st	Iri/5-FU/LV	BV
								2nd	Ox/5-FU/LV	-
**21**	m	73	right	yes	>2	HEP, PUL, LYM	UICC IV	2nd	Ox/5-FU/LV	BV
**22**	f	68	left	yes	>2	HEP, PUL, PER	UICC IV	3rd	Reg	-
**23**	m	61	rectum	yes	>2	HEP, LYM, PUL	UICC IV	1st	Iri/5-FU/LV	Cet
**24**	m	53	right	yes	2	HEP, LYM	UICC IV	1st	Iri/5-FU/LV	BV
**25**	m	63	rectum	yes	1	LYM	UICC IV	1st	Iri	-
**26**	f	45	left	yes	2	HEP, PER	UICC IV	2nd	Iri	Pan
**27**	m	66	left	yes	2	HEP, LYM	UICC IV	1st	Cap	BV

Ox=Oxaliplatin, Iri=Irinotecan, BV=Bevacizumab, Cet=Cetuximab, Pan=Panitumumab, Reg=Regorafenib, Cap=Capecitabine, Afl=Aflibercept, LV=Leucovorin, 5-FU=5-Fluorouracil, UICC=union international contre le cancer
