# Supplementary material for: Treatment monitoring in metastatic colorectal cancer patients by quantification and KRAS genotyping of circulating cell-free DNA
Source: PLoS One. 2017 Mar 22;12(3):e0174308. doi: 10.1371/journal.pone.0174308 (PMC5362218; doi:10.1371/journal.pone.0174308)
Supplement: S3 Table — (PDF) [file pone.0174308.s005.pdf]

ID	Sex	Age (years)	Primary tumor	Primary tumor resected	Metastases		Stage	Tissue *KRAS*-status	cfDNA *KRAS*-status
					Sites	Localization			
**28**	f	60	left+rectum	yes	>2	HEP, LYM, PUL	UICC IV	G13D	codon 12/13
**29**	m	52	rectum	yes	>2	HEP, LYM, PUL	UICC IV	G12R	wt
**30**	m	59	right	yes	1	HEP	UICC IV	G12C	wt
**31**	m	80	left	yes	1	HEP	UICC IV	G12V	codon 12/13
**32**	m	33	right	yes	1	HEP	UICC IV	G12V	codon 12/13
**33**	f	61	left	yes	1	PER	UICC IV	G13D	codon 12/13
**34**	m	65	rectum	yes	>2	HEP, LYM, PUL	UICC IV	G13D	codon 12/13
**35**	m	79	rectum	yes	>2	LYM, OSS, PUL	UICC IV	G13D	codon 12/13
**36**	m	43	right	yes	>2	HEP, LYM, PUL	UICC IV	G12S	codon 12/13
**37**	m	51	rectum	yes	2	LYM, PUL	UICC IV	G13D	codon 12/13
**38**	m	68	rectum	yes	1	HEP	UICC IV	G12A	codon 12/13
**39**	m	69	rectum	yes	>2	HEP, LYM, OSS, PUL	UICC IV	G12V	codon 12/13
**41**	m	80	rectum	yes	1	PUL	UICC IV	G13D	wt
**42**	f	57	rectum	yes	>2	BRA, OTH, PUL	UICC IV	A146T	A146T
**43**	m	72	left	yes	>2	OTH, PER, PUL	UICC IV	G12G	wt
**44**	f	79	right	yes	>2	LYM, PER, SPL	UICC IV	G12S	codon 12/13
**45**	f	58	right	yes	2	HEP, LYM	UICC IV	G12S	wt
**46**	f	73	right	no	1	HEP	UICC IV	G12R	codon 12/13
**47**	m	79	right	yes	1	HEP	UICC IV	G12D	codon 12/13
**48**	m	64	rectum	no	>2	HEP, LYM, PUL	UICC IV	G12D	codon 12/13
**49**	f	70	left+right	yes	>2	HEP, LYM, PER	UICC IV	G12C	codon 12/13
**50**	m	58	left	yes	>2	HEP, OSS, SPL	UICC IV	G12V	codon 12/13
**51**	m	53	right	yes	1	HEP	UICC IV	Q61R	Q61R

UICC=union international contre le cancer, wt=wild-type for tested hot-spots
